# Quality of Life and Psychopathology in Adults Who Underwent Hematopoietic Stem Cell Transplantation (HSCT) in Childhood: A Qualitative and Quantitative Analysis

**DOI:** 10.3389/fpsyg.2017.01316

**Published:** 2017-08-08

**Authors:** Francesco Sinatora, Annalisa Traverso, Silvia Zanato, Nicoletta Di Florio, Alessio Porreca, Marta Tremolada, Valentina Boscolo, Antonio Marzollo, Chiara Mainardi, Elisabetta Calore, Marta Pillon, Chiara Cattelan, Giuseppe Basso, Chiara Messina

**Affiliations:** ^1^Psychiatric Unit, Department of Women's and Children's Health, University Hospital of Padua Padua, Italy; ^2^Department of Developmental Psychology and Socialisation, University of Padua Padua, Italy; ^3^Haematology-Oncology Division, Department of Women's and Children's Health, University Hospital of Padua Padua, Italy

**Keywords:** HSCT, transplantation, pediatric survivors, narration, HSCT psychological sequelae, quality of life, adult psychopathology

## Abstract

**Background:** Patients who undergo pediatric Hematopoietic Stem Cell Transplantation (HSCT) may experience long-term psychological sequelae and poor Quality of Life (QoL) in adulthood. This study aimed to investigate subjective illness experience, QoL, and psychopathology in young adults who have survived pediatric HSCT.

**Method:** The study involved patients treated with HSCT in the Hematology-Oncology Department between 1984 and 2007. Psychopathology and QoL were investigated using the SCL-90-R and SF-36. Socio-demographic and medical information was also collected. Finally, participants were asked to write a brief composition about their experiences of illness and care. Qualitative analysis of the texts was performed using T-LAB, an instrument for text analysis that allows the user to highlight the occurrences and co-occurrences of lemma. Quantitative analyses were performed using non-parametric tests (Spearman correlations, Kruskal-Wallis and Mann-Whitney tests).

**Results:** Twenty-one patients (9 males) participated in the study. No significant distress was found on the SCL-90 Global Severity Index, but it was found on specific scales. On the SF-36, lower scores were reported on scales referring to bodily pain, general health, and physical and social functioning. All the measures were significantly (*p* < 0.05) associated with specific socio-demographic and medical variables (gender, type of pathology, type of HSCT, time elapsed between communication of the need to transplant and effective transplantation, and days of hospitalization). With regard to the narrative analyses, males focused on expressions related to the body and medical therapies, while females focused on people they met during treatment, family members, and donors. Low general health and treatment with autologous HSCT were associated with memories about chemotherapy, radiotherapy, and the body parts involved, while high general health was associated with expressions focused on gratitude (*V*-Test ± 1.96).

**Conclusion:** Pediatric HSCT survivors are more likely to experience psychological distress and low QoL in adulthood compared with the general population. These aspects, along with survivors' subjective illness experience, show differences according to specific medical and socio-demographic variables. Studies are needed in order to improve the care and long-term follow-up of these families.

## Introduction

Hematopoietic stem cell transplantation (HSCT) is an effective therapy for malignant and non-malignant blood disorders and diseases of the immune system, consisting of the transference of stem cells from bone marrow, peripheral blood, or cord blood (Edman et al., 2001; DeMarinis et al., 2009). The entire procedure can be carried out using the patient's body (autologous HSCT) or involving a healthy donor (allogeneic HSCT) who can be related or unrelated. The progressive refinement of intervention techniques has led to an increase in HSCTs and survival from childhood cancer. It is estimated that at least 50,000 transplants are carried out worldwide every year (Gratwohl et al., 2010). Effective treatment, supportive care, and a focus on patients' wellbeing have produced a rise in the long-term survival rate, from 70 to 90% of treated individuals (Langeveld et al., 2002).

HSCT is characterized by a complex medical nature that involves invasive medical procedures associated with extended hospitalizations, long periods of isolation, adverse side effects, risk of mortality or relapse, and long recovery periods (Rueda-Lara and Lopez-Patton, 2014). Patients face substantial changes in their physical functioning due to symptoms such as fatigue, dry mouth, loss of appetite, nausea, and mucositis (Rueda-Lara and Lopez-Patton, 2014). Even when the transplantation is carried out successfully, recipients are at risk of short- and long-term medical consequences such as chronic Graft-vs.-Host Disease (GvHD), infections, secondary malignancies, endocrine dysfunction, and different physical symptoms like pain, nausea, and fatigue (Lowe et al., 2007). It is estimated that at least 25% of long-term HSCT survivors are affected by chronic consequences (Socié et al., 2003). For this reason, although HSCT offers individuals higher life expectancies, it is a procedure that involves intense emotional distress. The long hospital stays, isolation, variation in medical status, invasive procedures, and side effects, including the mortality risk implicit in the procedure itself, all constitute significant physical and psychological stressors to which the recipients and their families are exposed (Niederbacher et al., 2012). Physical sequelae have been investigated in several follow-up studies (Majhail and Rizzo, 2013), but long-term burdens include the effect of the HSCT experience on emotional, psychological, and social development as well. Some authors have suggested that the patient's disease and transplantation can be conceptualized as a psychosocial transition that requires the individual to change his/her perspective of himself/herself and adjust to the world (Parkes, 1971; Mosher et al., 2009).

As survival rates increase it is necessary to direct attention to the psychosocial impact of the experience of the illness which, according to the literature, could persist beyond the moment of hospitalization and transplantation (Hjermstad et al., 1999, 2004; Bevans, 2010).

Many studies have highlighted the finding that HSCT has a significant impact upon Quality of Life (QoL) (Bevans et al., 2008; Pidala et al., 2009), due to its heterogeneous late effects and their influence on physical and psychological wellbeing (Socié et al., 2003; Majhail and Rizzo, 2013; Poloméni et al., 2016).

The Word Health Organization defines Quality of Life as a state of wellbeing rather than merely the absence of disease (WHO, 1948). This definition suggests a multi-dimensional approach that encompasses physical outcomes as well as life quality and perceived wellbeing. Moreover, the centrality of the subjective experience is underlined, as indicated in its reference to “individuals' perceptions of their position in life in the context of the culture and value system in which they live and in relation to their goals, standards, and concerns” (WHO, 1948, p. 153). The construct includes dimensions such as physical health, psychological state, levels of independence, social relationships, environmental features, and spiritual concerns (Niederbacher et al., 2012). These dimensions are particularly important in gaining a general understanding of the views of pediatric patients who have undergone HSCT, where physical outcomes are linked to emotional and psychological outcomes.

Studies have shown that QoL varies between individuals and over time (Hjermstad et al., 2004; Bieri et al., 2008). It is suggested that impairments are likely to begin prior to transplantation, as a consequence of either the disease itself or previous treatments. During HSCT the major influence on QoL is exerted by aspects of physical functioning and symptoms (Pidala et al., 2009). During the first year after HSCT and later, the effects on QoL are mainly observable in domains related to physical functioning, social relationships, and role function (Zeltzer et al., 2008; Bevans, 2010); this is probably due to the impact of long hospitalizations and subsequent limitations on activities on these areas (Vannatta et al., 1998). Furthermore, HSCT also affects family QoL, with recipients' close relatives reporting an increase in physical and psychological symptoms (Wulff-Burchfield et al., 2013). Some studies have shown that the majority of recipients return to basic levels of QoL after 1 year post-HSCT (Syriala et al., 1993; Wettergren et al., 1997; Pidala et al., 2009). Others have found that while patients' overall functional status might return to its baseline, their physical, role and social functions are likely to be significantly impaired compared with those of the general population (Hjermstad et al., 1999, 2004; Ness et al., 2005; Pemberger et al., 2005; Maunsell et al., 2006; Smith et al., 2010).

With regard to psychopathology, various studies have shown a higher incidence of symptoms in HSCT survivors, with distress, anxiety, and depression reported as present during all phases of HSCT (Bevans, 2010). The most common psychiatric diagnoses during transplantation were adjustment disorders, mood disorders, and anxiety disorders (Prieto et al., 2002). During hospitalizations especially, patients are likely to experience mood deterioration and show an increase in depressive symptoms (El-Jawahri et al., 2015). During the later phases of transplantation, uncertainty about relapse constitutes an additional stressor (Bevans, 2010). Although, some studies have suggested the presence of progressive improvements in psychological wellbeing after discharge (Syriala et al., 1993; McQuellon et al., 1997; Bevans et al., 2006; Schulz-Kindermann et al., 2007), others have highlighted a certain stability after this initial period (Syriala et al., 1993; McQuellon et al., 1997). Moreover, various studies have reported the presence of anxiety and depression in recipients up to 2 or 3 years after transplantation (Chang et al., 2005; Lee et al., 2005; Conner-Spady et al., 2006; Beglinger et al., 2007; Hoodin et al., 2013), thus highlighting the fact that despite initial improvements, compared with non-cancer groups survivors are more likely to experience long-term impairments in emotional functioning (Pidala et al., 2009; Bevans, 2010; Zanato et al., 2017) even in adulthood (Sanders et al., 2010). In particular, females are more at risk of experiencing psychological dysfunction through anxiety and depressive symptoms (Zeltzer et al., 1997; Michel et al., 2010; Ahomäki et al., 2015).

Taken together, these findings suggest that individuals exposed to HSCT in childhood are at higher risk of experiencing psychological distress and poor QoL during adulthood. While this is generally recognized to be the case, many psychological issues are still less well understood. From this point of view, patients' narrations of their own experience could help to shed light on the burden of receiving a cancer diagnosis, undergoing infusions, and the process of restructuring one's life after HSCT. The theoretical and epistemological approach of narrations permits one to explore what remains in the minds and memories of survivors, giving them the ability to tell their stories in their own words. The underlying assumption is that the construction of meanings about lived experiences originates in narrative acts. Narrative acts constitute a possible way for individuals to let others know their story and themselves, essentially, in accordance with the theory that the construction of meanings corresponds to the construction of the Self (Bruner, 1990; Villegas, 1993; Vezzani, 1998). Longitudinal assessment using quantitative and qualitative methods could be useful for various reasons. First, taking survivors' perspectives into account could help researchers to reach a better and more complete understanding of their experience (Bevans, 2010). Second, continuous screening could help to identify risk factors for psychological distress, facilitating and guiding appropriate interventions, and ultimately improving outcomes (Rueda-Lara and Lopez-Patton, 2014).

To our knowledge, only a few studies have adopted qualitative approaches to study long-term consequences among HCST survivors (Beeken et al., 2011; Lahaye et al., 2017). In order to help fill this gap in the literature, the aim of the present study was to investigate QoL and psychological wellbeing in young adults who have survived HSCT performed at pediatric age, from both a quantitative and qualitative point of view. More specifically, the study aimed to: (a) investigate long-term QoL perceptions after transplantation; (b) assess the frequency of psychopathology in HSCT survivors; (c) investigate the subjective experience of disease and HSCT through narrations; and, (d) determine whether specific medical and socio-demographic variables are associated with QoL and psychopathology.

In relation to QoL, we hypothesize that survivors are more inclined to experience difficulties in physical, role, and social functions, while in relation to psychopathology we hypothesize that they are more likely to experience long-term impairments in emotional functioning. Regarding specific medical and socio-demographic variables, we expect allogeneic-HSCT survivors to report lower physical mean scores on the SF-36, and hypothesize that females and unrelated allogeneic donor transplant recipients are more at risk of developing psychopathology, in accordance with the literature.

## Materials and methods

### Subjects and procedures

The research involved adults who had undergone HSCT treatment during childhood. The inclusion criteria were:

- Being of adult age (>18 years)
- Having undergone HSCT treatment at pediatric age
- At least 5 years having elapsed since HSCT (the period that the literature identifies as being necessary to be considered a survivor)
- Being able to read, speak, write, and understand Italian
- The absence of intellectual disability that could prevent correct understanding and completion of the task
- Being of stable health.

In 2015, 161 adult HSCT survivors were drawn from the Local registry of Transplantation of Pediatric Onco-Hematology at the University Hospital of Padua. All patients had been treated at the Pediatric Hematology-Oncology Division, Department of Women's and Children's Health, University of Padua, and had undergone transplantation between 1983 and 2006. Annually, around 150 new oncological patients are referred to this institution and around 40 HSCTs are performed.

The survivors were contacted by a preliminary phone call from trained personnel, explaining the objectives of the study. Those who initially agreed to participate in the research (*n* = 44) were sent, by post or e-mail, a folder containing a brief description of the study, an informed consent form, and a self-report questionnaire. Twenty-one of them went on to participate in the study, 18 of whom (11 female) participated in all the steps, including the production of text for T-Lab.

Information regarding participants' history was collected from their medical sheets by medical staff. Socio-demographic information was collected during a 30-min structured interview carried out over the phone by trained personnel. Table 1 shows the socio-demographic and medical information of the participants. The study was carried out after receiving approval from the Ethical Committee of the Department of Women's and Children's Health, Padua in March 2015.

**Table 1:** Socio-demographic and medical characteristics of HSCT survivors (*n* = 21).

	**N (%)**	**M (*SD*)**	**Range**
**GENDER**			
Male	9 (42.9%)		
Female	12 (57.1%)		
**EDUCATION (YEARS OF SCHOOLING)**			
8	1 (4.8%)		
13–16	14 (66.7%)		
18	4 (19.0%)		
Other	2 (9.5%)		
**RELATIONSHIP STATUS**			
Single	19 (90.5%)		
Engaged or married	2 (9.5%)		
**UNDERLYING PATHOLOGY**			
Leukemia	12 (57.1%)		
Lymphomas and solid tumors	5 (23.8%)		
Others^*^	4 (19.0%)		
**TYPE OF HSCT**			
Autologous	8 (38.1%)		
Allogeneic unrelated	6 (28.6%)		
Allogeneic related and/or haploidentical	7 (33.3%)		
Intensification with double HSCT	1 (5%)		
**AGE (YEARS)**			
Current		26.70 (5.16)	19.7-36.79
At HSCT		9.27 (4.09)	1.65-16.37
Time elapsed from HSCT (years)		17.42 (6.84)	8.59-32.04
Days of hospitalization in Transplantation Unit		59.61 (52.44)	20-180
Days of hospitalization after HSCT		14.76 (31.08)	0-141

* *Mucopolisaccharidoses, Cooley's anemia, Fanconi anemia*.

## Tools

### Semi-structured interview and checklist for the collection of case histories

Relevant demographic, medical, and psychological data was investigated using information collected from subjects' medical sheets. The socio-demographic information to be investigated was obtained from structured interviews conducted by trained personnel, and included specifically: age, gender, and education of the child/adolescent, as well as family composition, socioeconomic status, immigration status, and distance between the subject's residence and the medical center. The medical variables included underlying pathology, length of isolation associated with the transplantation, and presence of complications. Table 1 shows the medical and socio-demographic information.

The psychological variables included the spontaneous request for psychological help and a history of previous psychological counseling. Nineteen percent of subjects reported having previously sought psychological counseling, whereas none reported any current request for psychological help or the current use of psychopharmacological treatment. The majority of participants declared that the medical information they had received about the procedure had been inadequate (65%) or only partially adequate (20%). The remainder (15%) did not have an opinion about this aspect. Parental support was perceived to have been completely adequate (66.7%) or adequate (28.6%).

### 36-item short form health survey (SF-36)

The *36-Item Short Form Health Survey* (SF-36; Ware, 1993) is a 36-item self-report measure aimed at investigating perceived health, functioning, and quality of life. The questionnaire has two summary scales and eight individual subscales: physical functioning, role limitations resulting from physical health problems, role limitations resulting from emotional problems, social functioning, mental health, energy and vitality, bodily pain, and general health perception. Scores on each scale range from 0 to 100, with higher scores indicating better QoL (Ware and Sherbourne, 1992). For the purposes of the present study the Italian version of the instrument was adopted (Apolone and Mosconi, 1998). The instrument has proved to be valid, reliable, and applicable across age, gender, and disease (Apolone and Mosconi, 1998).

### Symptom Check-list-90-R (SCL-90-R)

The *Symptom Checklist-90 Revised* (*SCL-90-R;* Derogatis, 1977; Italian version by Sarno et al., 2011) is a 90-item self-report questionnaire aimed at assessing the presence of psychological distress and a range of psychopathological symptoms. The scores lead to different symptom dimensions (Somatization, Obsessive-compulsive, Interpersonal sensitivity, Depression, Anxiety, Hostility, Phobic anxiety, Paranoid ideation, Sleep Disorders, and Psychoticism), and three global distress indexes. The latter refer to global psychological distress status (Global Severity Index–GSI), the total number of symptoms reported (Positive Symptom Total–PST) and the intensity of reported distress (Positive Symptom Distress Index–PSDI). By converting the raw scores into T-scores, it is possible to compare individual values with normative cut-off values. Each item's score can be interpreted as being below, within, above or definitely above the average scores of the normative sample, thus indicating the presence of severe symptomatology.

### T-lab

In order to identify qualitative aspects of the disease experience, participants were asked to write a brief text describing their illness and transplant experience. The task was: “*We ask you to describe, freely, your experience of the disease that you had to deal with*.” Patients' texts were explored using narrative analysis, a method that aims to capture the meanings within the narration and the semantic cores that recur most frequently in the texts. Analysis of the narrations was performed by T-lab (Ver. 8.1.4; Lancia, 2012). This text analysis software comprises several instruments, including linguistic, statistical, and graphic tools. The instrument can analyse both quantitative and qualitative data.

A coding-line with codified variables was assigned to each single text in order to maintain its specificity. The variables used for the coding-lines were: gender, type of disease (leukemias, lymphomas, and solid tumors, others), type of HSCT (autologous, allogeneic from unrelated donor, allogeneic from related donor), time elapsed from diagnosis to HSCT (≤1 year, >1 year), and T scores on the general health scale of the SF-36 (<2SD, ≥2SD).

After this first phase, all the texts were combined to form a single, long script that was “cleaned” by removing words without meaning, such as articles or prepositions, in order to condense meanings (Angioi et al., 2008) into a *corpus*.

Words with different meanings or full of meanings (compound words, phrasal verbs, idioms) were then disambiguated and assigned to a *lemma*. A lemma is a headword that designates words referring to the same semantic area. During the processes of exclusion and disambiguation, four non-independent referees reached agreement of at least 75%. Correspondence analysis, Multiple Correspondence Analysis, Co-Word Analysis, Concept Mapping and Specificity analysis were run on the lemma obtained (see Appendix for more details on the procedure). Analyses were initially run on the Italian lemma and subsequently on the lemma translated into English. The results showed no substantial differences.

### Statistical analyses

Data was analyzed using IBM SPSS statistics version 23 (IBM Corporation, Armonk NY, USA)^1^. Given the small number of subjects who finally agreed to take part in the research, preliminary comparative analyses were run between participants and non-participants in order to see whether the two groups differed according to age, gender, underlying pathology, or type of transplantation. A chi-square analysis was applied to explore the variables gender, underlying pathology (leukemias vs. lymphomas and solid tumors vs. others), and type of transplantation (autologous vs. allogeneic unrelated vs. allogeneic related and/or haploidentical), while the Mann-Whitney test was applied to the variable age. Descriptive analyses (frequencies, mean scores, standard deviations) were performed on the SF-36 and SCL90-R scores in order to assess the percentages of survivors belonging to a clinical category.

Non-parametric tests were then run to identify possible significant differences on QoL and psychopathology scales relating to socio-demographic or illness variables. Spearman correlations were run for one level-variables, while Kruskal-Wallis or Mann-Whitney tests were used with ordinal independent variables. Finally, Multiple Correspondence Analysis, Co-Word Analysis and Concept Mapping were performed on the narrative text (as described above).

## Results

### Comparison between participants and non-participants

No differences were found for gender [*X*^2^(1, N = 161) = 1.30, *p* > 0.05], type of pathology [*X*^2^(2, N = 161) = 2.70, *p* > 0.05], or type of transplant [*X*^2^(2, N = 160) = 1.38, *p* > 0.05].

The two groups differed with respect to age (*Z* = −2.16, *p* = 0.03), with participants (*M* = 9.27 years; *SD* = 4.09) being younger than non-participants (*M* = 11.44 years; *SD* = 4.93) at the time of transplantation.

### QoL in HSCT survivors

Table 2 presents the Quality of Life scores at ≤ 2 SD from the norms of the various subscales. Adult HSCT survivors showed poor QoL on the scales of bodily pain, general health perception, and physical functioning. Impairments in social functioning were reported by 23.8% of survivors, whereas a smaller percentage reported problems in other areas, such as mental health, role limitations due to physical or emotional problems, and energy/vitality.

**Table 2:** Subjects with clinical scores on the SF-36 scales (*n* = 21).

	**Clinical (≤2 *SD*)**	
	**N°**	**%**
Physical functioning	8	38.1
Role limitations resulting from physical health problems	2	9.5
Mental health	2	9.5
Role limitations resulting from emotional problems	4	19.0
Energy/vitality	1	4.8
Social functioning	5	23.8
Bodily pain	12	57.1
General health perception	12	57.1

### Psychopathology in HSCT survivors

A detection of caseness of psychopathology indexes adopting SCL-90 was run and is shown in Table 3. From the SCL-90-R scores, we can see that only one person exceeded the normative cut-off values for global psychological distress status (GSI Index). Looking at the raw scores, some of the survivors showed clinically significant difficulties in terms of sleep disorders (28.6%), obsessive-compulsive symptoms (23.8%), and interpersonal sensitivity (23.8%), while recording lower clinical-level scores on the other scales (ranging from zero to 14.3%).

**Table 3:** SCL90-R scores distribution (*n* = 21).

**SCL90-R scales**	***Clinical***	
	**N°**	**%**
Somatization	4	19.0
Obsessive-compulsive	5	23.8
Interpersonal sensitivity	5	23.8
Depression	3	14.3
Anxiety	3	14.3
Hostility	2	9.5
Phobic anxiety	0	0
Paranoid ideation	3	14.3
Psychoticism	2	9.5
Sleep disorders	6	28.6
Global Severity Index	1	4.8

### Socio-demographic variables and QoL

With respect to gender, females reported significantly higher scores (Mean rank = 13.54) than those of males (Mean rank = 7.61) on the mental health scale (*U* = 23.5; *p* = 0.028).

For the variable type of HSCT, adults who experienced autologous transplantations at pediatric age reported significant lower scores on the scales of inherent role limitations resulting from emotional problems [χ^2^(2) = 8.04; *p* = 0.018] and social functioning [χ^2^(2) = 7.45; *p* = 0.024]. Other pathology variables such as time elapsed between communication of the need to transplant and effective transplantation, type of pathology, days of hospitalization in HSCT Unit or after HSCT, age at HCST, and years from HSCT to assessment, were not significantly associated with adult survivors' scores on the QoL scales (*p* > 0.05).

### Differences in psychopathology mean indexes according to medical and socio-demographic variables

Males reported more psychopathology than females did on the following scales: obsessive-compulsive (*U* = 23; *p* = 0.028), depression (*U* = 21.5; *p* = 0.018), phobic anxiety (*U* = 25; *p* = 0.041), paranoia (*U* = 20.5; *p* = 0.015), psychoticism (*U* = 11; *p* = 0.003), sleep disorders (*U* = 15; *p* = 0.004), and the Global Severity Index (*U* = 15.50; *p* = 0.010).

A significant mean rank difference was discovered for type of HSCT on the following psychopathology scales: depression [χ^2^(2) = 8.04; *p* = 0.018], somatization [χ^2^(2) = 7.45; *p* = 0.024], psychoticism [χ^2^(2) = 6.97; *p* = 0.031], and the Global Score Index [χ^2^(2) = 7.49; *p* = 0.024]. Adults who experienced autologous transplantations at pediatric age reported higher scores for depression (Mean rank = 15.38), somatization (Mean rank = 13.69), psychoticism (Mean rank = 14.38), and on the Global Score Index (Mean rank = 14.69) compared with adults who experienced allogeneic (both related and unrelated) HSCT.

The variable time elapsed between communication of the need to transplant and effective transplantation (1–6 months, 6–12 months, 1–3 years, not known) also impacted significantly on adult survivors' current psychopathology, on the following scales specifically: interpersonal sensitivity [χ^2^(3) = 8.37; *p* = 0.039], depression [χ^2^(3) = 8.4; *p* = 0.038], psychoticism [χ^2^(3) = 9.29; *p* = 0.026], phobic anxiety [χ^2^(3) = 7.95; *p* = 0.047], and sleep disorders [χ^2^(3) = 8.03; *p* = 0.046]. Patients who had waited 6–12 months before HSCT reported higher scores on these scales than did subjects who had waited either less than 6 months or more than 12 months.

Neither age at HSCT nor years elapsed since HSCT had any significant impact on any of the psychopathology scales (*p* > 0.05). Spearman's correlations showed a significant association between days of hospitalization in HSCT Unit and phobic anxiety (*r* = −0.44; *p* = 0.04). Days of hospitalization after HSCT was significantly related to the depression scale (*r* = −0.44; *p* = 0.04). Type of pathology (leukemias, solid tumors, others) had a significant impact on several psychopathology scales: obsessive-compulsive [χ^2^(2) = 7.49; *p* = 0.024], depression [χ^2^(2) = 7.49; *p* = 0.024], anxiety [χ^2^(2) = 7.49; *p* = 0.024], paranoid ideation [χ^2^(2) = 7.49; *p* = 0.024], and the Global Severity Index [χ^2^(2) = 7.49; *p* = 0.024], with survivors with a solid tumor showing more psychopathology symptoms than those in the other two categories.

### Subjective experience of disease and HSCT through narrations

The 18 brief compositions constitute an original corpus of 7026 occurrences, 1801 words, 1293 lemma, and 1131 hapax (Lancia, 2012). After the cleaning process, we obtained a corpus of 855 lemma.

### Co-word analysis and concept mapping

**Figure 1:**
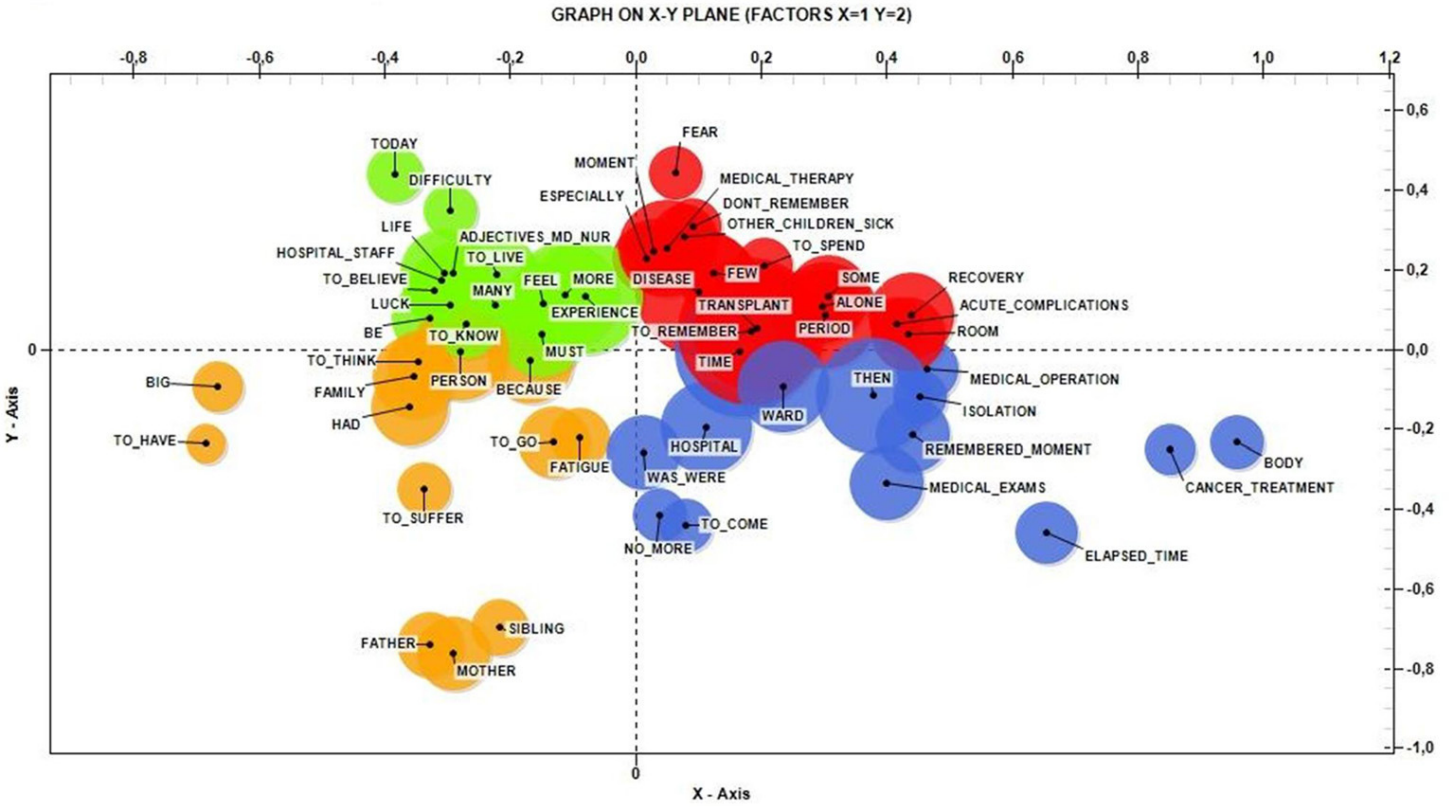
Co-word analysis and concept mapping.

After setting a threshold of 10 occurrences *per* lemma, the software selected a group of 92 lemma. We examined the 62 most significant lemma (Table 4), whose semantic areas in the corpus are represented in Figure 1.

**Table 4:** Significant lemma from T-lab Co-Word Analysis and Concept Mapping (*N* = 18).

**F1 Test value**				**F2 Test value**			
**Pole (−)**	**Test-V**	**Pole (+)**	**Test-V**	**Pole (−)**	**Test-V**	**Pole (+)**	**Test-V**
Big	−5.13	Body	7.42	Mother	−9.75	Today	4.08
To_Have	−4.75	Cancer_Treatment	7.13	Father	−8.82	Fear	3.88
Family	−4.61	Elapsed_Time	6.79	Sibling	−7.24	Medical_Therapy	3.73
Had	−4.53	Then	6.51	With	−6.52	Difficulty	3.41
Be	−4.52	Recovery	5.88	Always	−4.90	Other_Children_	3.19
Life	−4.36	Room	5.82	Elapsed_Time	−4.75	Don't_Remember	3.11
Person	−4.26	Remembered_Moment	5.48	To_Come	−4.45	Moment	2.84
To_Think	−4.16	Period	5.05	Medical_Exams	−4.03	Life	2.77
Father	−3.93	Medical_Exams	4.84	No_More	−3.91	Especially	2.66
Mother	−3.73	Medical_Operation	4.47	To_Suffer	−3.62	Disease	2.61
Luck	−3.70	Acute_Complication	4.11	Was_Were	−3.55	Few	2.56
Today	−3.57	Isolation	4.10	Hospital	−3.04	To_Live	2.55
To_Suffer	−3.52	Some	4.07	To_Go	−2.99	More	2.37
To_Believe	−3.52	To_Remember	3.73	Remembered_Moment	−2.67	Experience	2.27
Always	−3.45	Alone	3.63	Fatigue	−2.49	Adjectives_Md_N^a^	2.18
To_Know	−3.32	Transplant	3.50	Cancer_Treatment	−2.09	To_Spend	2.15
Adjectives_Md_N^a^	−3.32	Ward	3.35				
Hospital_Staff	−3.23	Time	3.14				
To_Live	−2.99	To_Spend	2.09				
Difficulty	−2.90						
Because	−2.50						
Many	−2.50						
With	−2.47						
Sibling	−2.26						
Must	−2.18						
Feel	−2.15						

a *Positive adjectives to describe doctors and nurses*.

The lemma Big, Body, Mother, and Today were the ones with higher statistical weight.

### Correspondence analysis

Setting the default threshold of 4 occurrences per lemma, we obtained 234 lemma. Table 5 shows the lemma most frequently used by male and female survivors.

**Table 5:** Lemma used by male and female survivors (*N* = 18).

**Female**		**Male**	
**Word**	**Test-V**	**Word**	**Test-V**
Mother	3.68	Cancer_Treatment	3.58
Person	3.47	Medical_Operation	3.21
To_Say	3.1	Especially	3.08
To_Go	2.83	Due_To	2.94
Was_Were	2.59	Without	2.93
Father	2.58	To_Force	2.68
Sibling	2.31	Fear	2.64
Md^a^	1.96	Medical_Therapy	2.57
		Period	2.52
		To_Begin	2.41
		To_Believe	2.40
		Drug	2.25
		Medical_Exams	2.17
		Negativity	2.11
		Stress	2.11
		Where	2.11
		Too_Much	1.96
		Other_Disease	1.96

a *Medical Doctors*.

**Figure 2:**
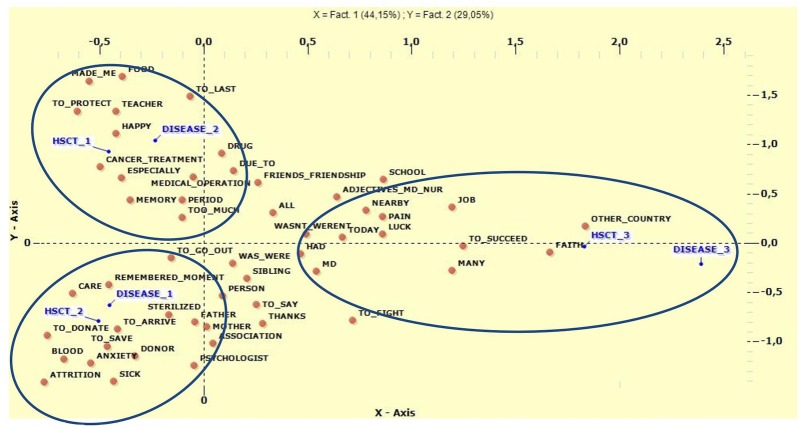
Multiple Correspondence Analysis between type of disease and type of HSCT. HSCTl, Autologous Transplant; HSCT2, Allogeneic un-related donor; HSCT3, Allogeneic related donor; Disease1; Leukemias; Disease2, Lymphomas and solid tumors; Disease3, Other disease.

First, we investigated whether specific lemma were associated with the variables underlying disease and type of HSCT. We found an association between leukemias and HSCT from unrelated donors (*V*-Test F2 Disease_1 = −8.23; HSCT_2 = −8.47). These variables were associated with lemma that referred to human subjects close to the narrator, such as Mother, Donor, Father, Person, and sibling. As for lymphomas, solid tumors, and autologous transplants (*V*-Test F2 Disease_2 = 9.39; HSCT_1 = 8.87), the most frequently used lemma were concerned with the period of care and illness (Period, Due To, To Last, Memory, Medical Operation, Cancer Treatment, Drugs). For the last group, other underlying diseases (Cooley's anemia, Fanconi anemia, Mucopolysaccharidosis) and allogeneic transplant from related donors (*V*-Test F1 HSCT_3 = 11.77; Disease_3 = 11.7) the lemma most used recalled luck at having come to Padua (Other Country) and gratitude toward professional figures (Medical Doctor, Positive Adjectives to describe Doctors and Nurses). Multiple Correspondence Analyses are presented in Figure 2, while the statistical details are reported in Table 6.

**Table 6:** Lemma frequently used for Multiple Correspondence Analysis between type of disease and type of HSCT.

**F1 Test Value**				**F2 Test Value**			
**Pole (−)**	**Test-V**	**Pole (+)**	**Test-V**	**Pole (−)**	**Test-V**	**Pole (+)**	**Test-V**
*Allogeneic unrelated donor*	−5.43	*Allogeneic related donor*	11.77	*Leukemias*	−8.24	*Lymphomas and solid tumors*	9.40
*Leukemias*	−5.96	*Other disease*	11.71	*Allogeneic unrelated donor*	−8.48	*Autologous transplant*	8.87
*Autologous transplant*	−4.36	Many	4.14	Mother	−4.12	Food	3.79
*Lymphomas and solid tumors*	−2.10	Other_Country	4.10	Donor	−4.29	Made_Me	3.79
Remembered_moment	−2.52	Faith	3.32	Father	−3.07	Period	3.55
		Md^a^	3.26	Person	−3.44	Due_To	3.51
		Luck	3.22	To_Say	−3.67	To_Last	2.98
		Job	2.92	Sterilized	−2.04	Memory	2.94
		School	2.82	To_Donate	−2.06	Especially	2.94
		Adjectives_md_n^b^	2.79	To_Fight	−2.08	Too_Much	2.85
		To_succeed	2.79	Sibling	−2.08	Teacher	2.68
		All	2.22	Care	−2.17	To_Protect	2.68
		Nearby	2.20	Thanks	−2.21	Medical_Operation	2.65
		Wasn't_weren't	2.19	Association	−2.27	Cancer_Treatment	2.56
		Had	2.13	Remembered_Moment	−2.30	Happy	2.49
		Today	2.11	Was_Were	−2.34	Drug	2.24
		Pain	2.01	To_Save	−2.38	Friends_Friendship	2.15
				To_Arrive	−8.24	All	2.08
				Anxiety	−8.48		
				Attrition	−4.12		
				Blood	−4.29		
				Psychologist	−3.07		
				To_Go	−3.44		
				Sick	−3.67		

a *Medical Doctors*.

b *Positive adjectives to describe doctors and nurses*.

**Figure 3:**
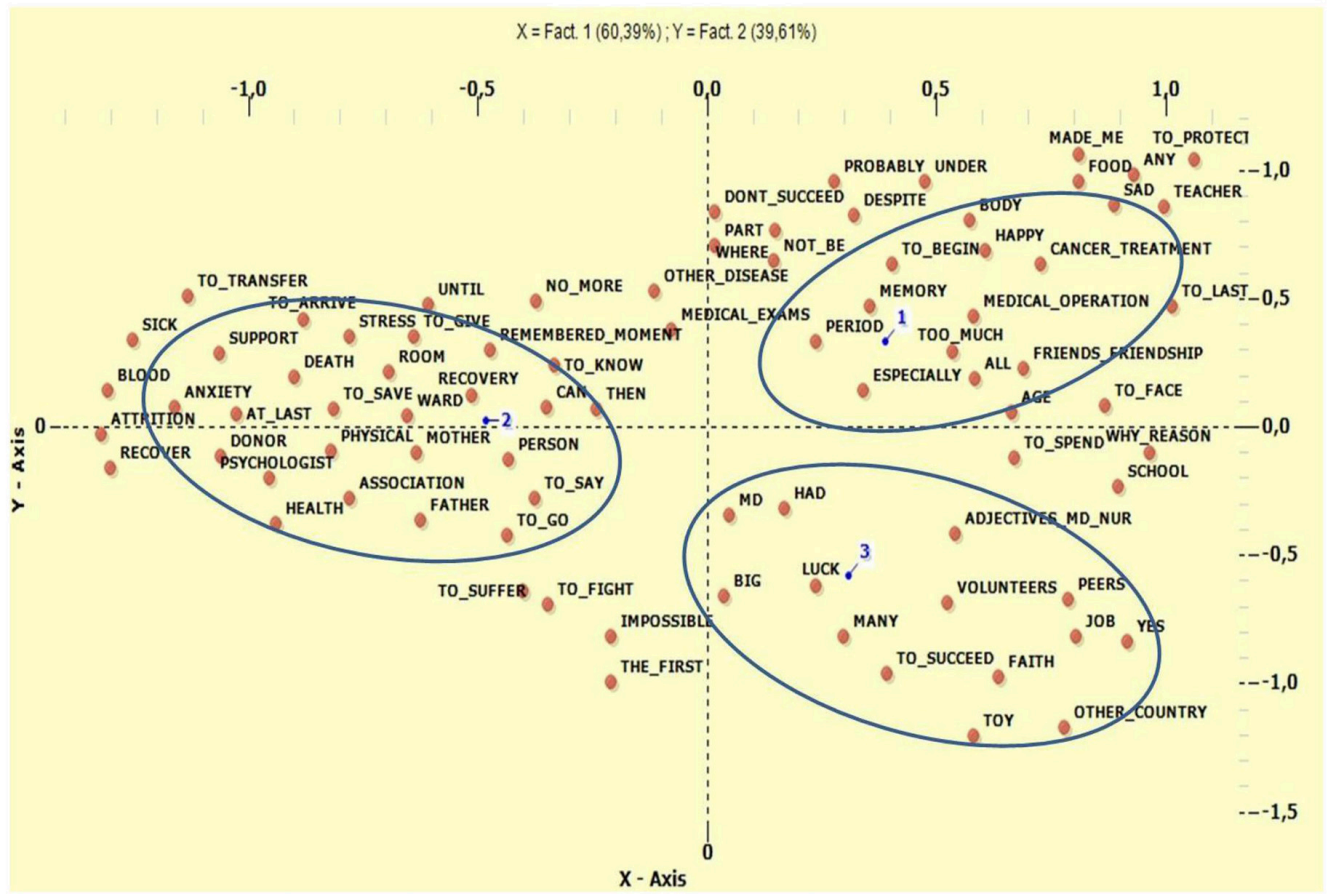
Correspondences Analysis for type of HSCT. 1, Autologous Transplant; 2, Allogeneic un-related donor; 3, Allogeneic related donor.

Figure 3 shows the differences in narrations with respect to type of HSCT. When considering only this variable, patients treated with autologous transplant (1 in Figure 3) used lemma that recalled the entire experience of HSCT and its invasiveness (Cancer Treatment, Body, Too Much, Memory, and Medical Operation). For survivors of HSCT from an unrelated donor, the lemma most often used referred to relational and physical aspects linked to hospitalization (Donor, Remembered Moment, Physical, Ward, Mother, and Father). In the group who underwent allogeneic transplant from a related donor, the most frequently used lemma were connected to the playful and relational characteristics of the “hosting” hospital (Toy, Positive Adjectives to describe Doctors and Nurses, Other Country, Faith, Luck, and Volunteers). All these results are shown in Table 7.

**Table 7:** Lemma frequently used for type of HSCT.

**Autologous transplant**		**Allogeneic unrelated donor**		**Allogeneic related donor**	
**Word**	**Test-V**	**Word**	**Test-V**	**Word**	**Test-V**
Cancer_treatment	4.09	Donor	3.83	Toy	4.74
Body	3.87	Remembered_moment	3.08	Adjectives_Md_Nur^a^	3.18
Too_Much	3.43	Physical	2.88	Other_country	3.10
Memory	2.79	Ward	2.88	Many	3.00
All	2.69	Mother	2.84	Job	2.61
Medical_operation	2.57	Death	2.59	Yes	2.53
Friends_friendship	2.28	To_Arrive	2.59	Faith	2.53
Happy	2.09	Support	2.59	Luck	2.49
Period	2.03	Room	2.57	Volunteers	2.21
Especially	2.01	Recovery	2.42	Peers	2.21
Normal	2	Person	2.34	To_succeed	2.02
To_begin	2	Can	2.23	Md^b^	2.00
		Father	2.21		
		Stress	2.03		
		To_Save	2.03		
		Psychologist	2.03		
		To_Say	1.97		
		Then	1.96		

a *Positive adjectives to describe doctors and nurses*.

b *Medical Doctors*.

**Figure 4:**
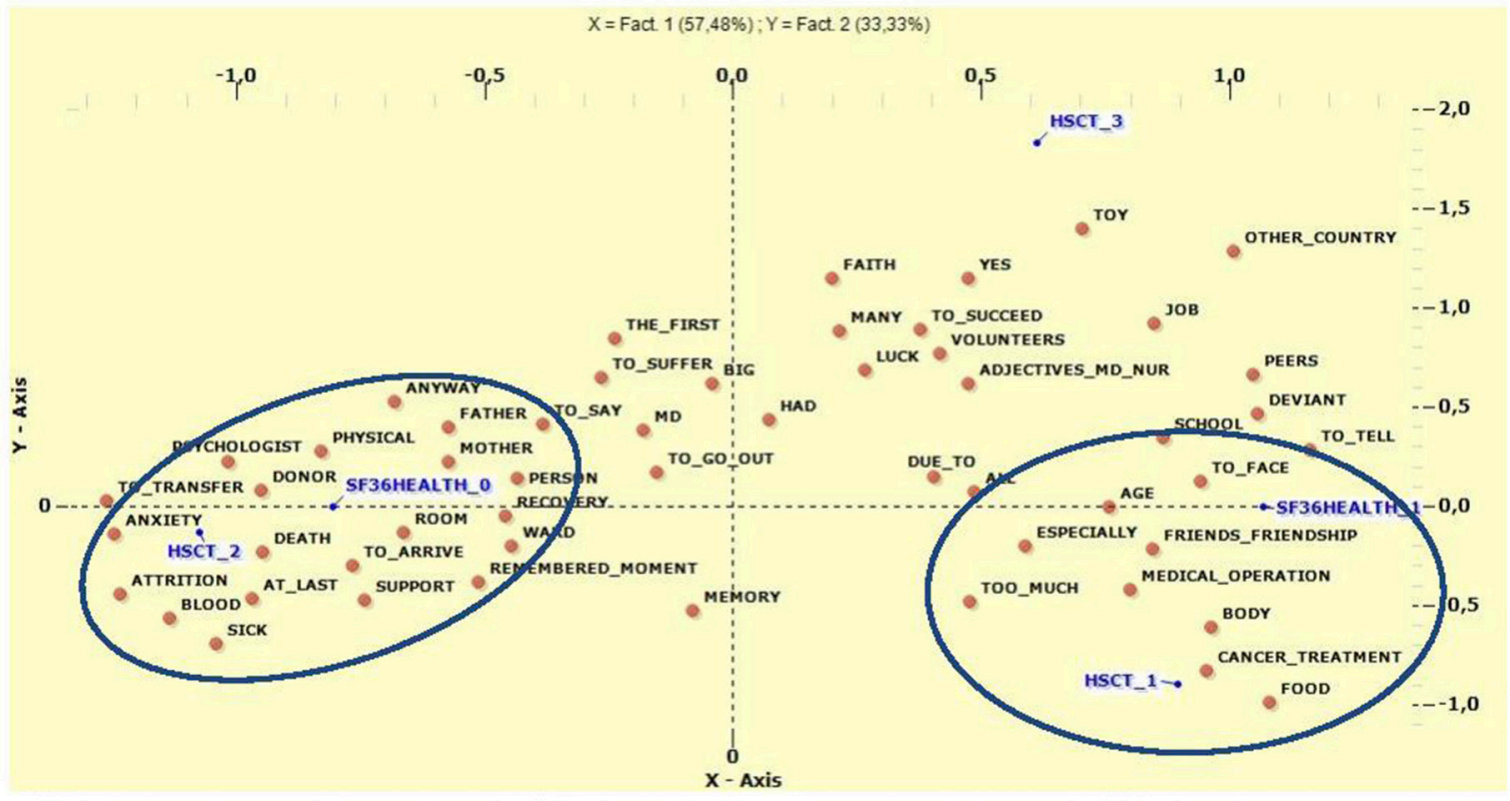
Multiple Correspondence Analysis between type of HSCT and General Health of SF-36. HSCT1, Autologous Transplant; HSCT2, Allogeneic un-related donor; HSCT3, Allogeneic related donor; SF3HEALTH_O, Scores >2SD; SF3HEALTH_1, Scores ≤ SD.

Multiple Correspondence Analysis was subsequently conducted in order to identify the most relevant lemma correlated with general health on the SF-36 and type of HSCT (Figure 4). We crossed factors F1 and F2, which explained most of the variance. In the negative polarity of the X axis, significant associations were found between HSCT from an unrelated donor (*V*-Test = −11.5629), higher scores on the SF-36 (*V*-Test = −11.6349), and lemma referring to people providing support during the traumatic experience (Mother, Donor, Father, Psychologist, Support, Anxiety). In relation to the positive polarity, we found associations between' lower SF-36 scores (*V*-Test = 11.6349) and the variables autologous transplant (*V*-Test = 8.5343), allogeneic transplant from related donor (*V*-Test = 3.9379), and lemma suggesting the strong impact of medical therapies (Too Much, Cancer Treatment, Body, and Medical Operation). Regarding F2, in the positive polarity of the Y axis we found associations between the variable allogeneic transplant from related donor (*V*-Test = 11.7905) and keywords referring to the novelty and the care associated with a “foreign” hospital (Toy, Other Country, Positive Adjectives to describe Doctors and Nurses, Volunteers, Luck, and To Succeed). Looking at the negative polarity of the ordinate, we found associations between the variable autologous transplant (*V*-Test = −8.5164) and the lemma of Memory, Body, and Cancer Treatment. The results are shown in Table 8.

**Table 8:** Lemma frequently used for Multiple Correspondence Analysis between General Health SF36 and type of HSCT.

**F1 Test value**				**F2 Test value**			
**Pole (−)**	**Test-V**	**Pole (+)**	**Test-V**	**Pole (−)**	**Test-V**	**Pole (+)**	**Test-V**
*Allogeneic unrelated donor*	−11.56	*Sf36health_1*	11.63	*Autologous transplant*	−8.52	*Allogeneic related donor*	11.79
*Sf36health_0*	−11.63	*Autologous transplant*	8.53	Memory	−2.37	Toy	4.67
Mother	−3.01	*Allogeneic related donor*	3.94	Body	−2.74	Many	3.08
Donor	−3.54	Too_Much	3.57	Cancer_Treatment	−2.88	Other_Country	2.88
To_Go	−2.05	Cancer_Treatment	3.31			Adjectives_Md_N^a^	2.72
Father	−2.13	All	3.25			Luck	2.58
Anxiety	−2.18	Body	3.11			Md^b^	2.34
Attrition	−2.18	School	2.88			Faith	2.30
Blood	−2.18	Peers	2.77			Yes	2.30
To_Transfer	−2.18	To_Face	2.66			Job	2.26
Recovery	−2.26	Food	2.55			The_First	2.09
Psychologist	−2.28	Friends_Friendship	2.53			To_Suffer	2.07
Support	−2.30	Medical_Operation	2.48			Big	2.07
To_Say	−2.34	Age	2.40			Volunteers	2.05
Remembered_Moment	−2.36	Toy	2.33			Had	2.01
At_Last	−2.44	Especially	2.28			To_Succeed	2.00
Recovery	−2.44	Any	2.28				
Person	−2.49	Other_Country	2.25				
Ward	−11.56	Deviant	11.63				
Sick	−11.63	To_Tell	8.53				
Death	−3.01	Normal	3.94				
To_Arrive	−3.54	Job	3.57				
Room	−2.05	Adjectives_Md_N^a^	3.31				
Physical	−2.13	Due_To	3.25				

a *Positive adjectives to describe doctors and nurses*.

b *Medical Doctors*.

**Figure 5:**
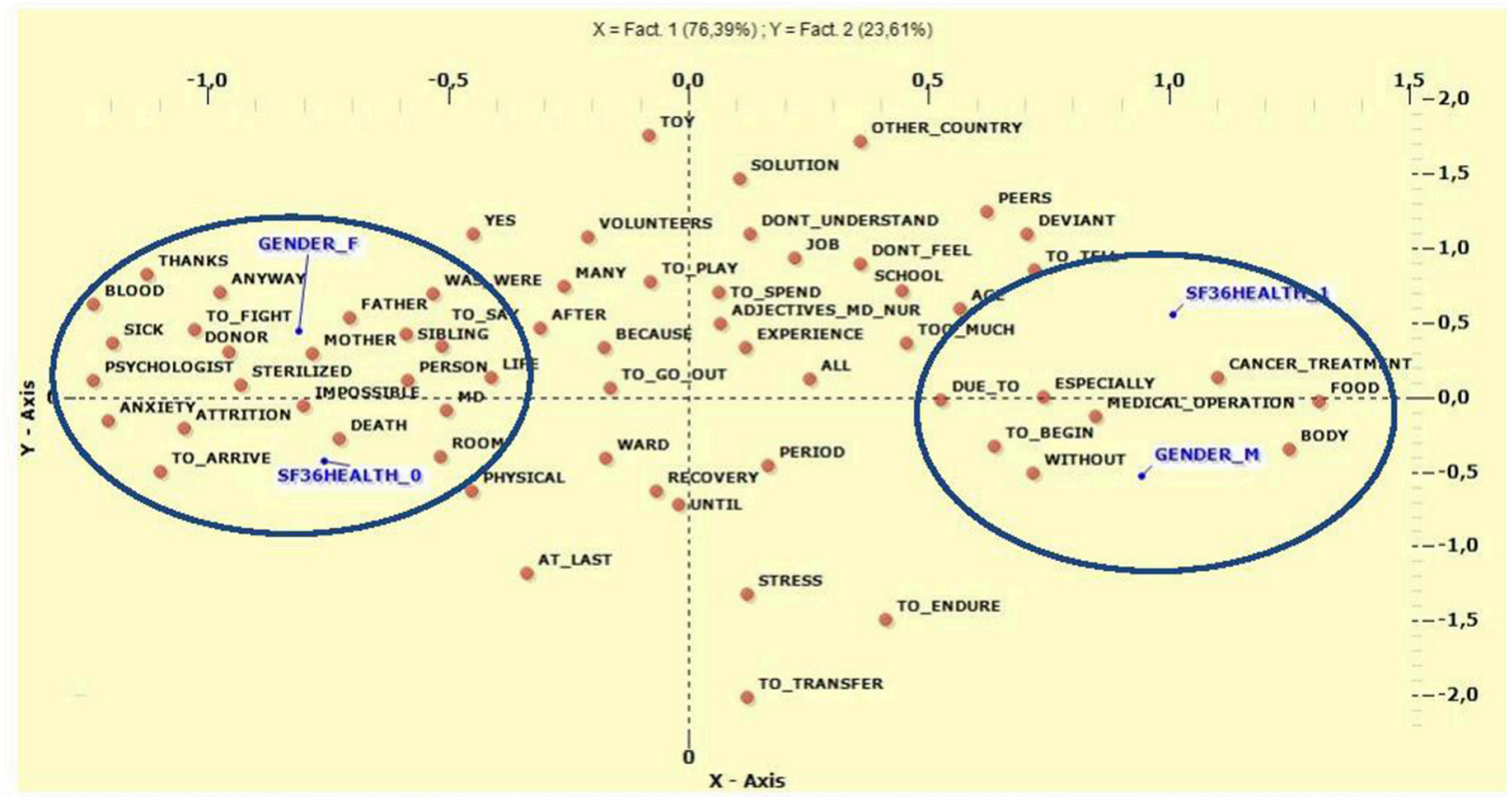
Multiple Correspondence Analysis between General Health SF36 and Gender. Gender_F, Female; Gender_M, Male; SF3HEALTH_O Scores >2SD; SF3HEALTH_1, Scores ≤ 2SD.

Finally, Multiple Correspondence Analysis was run to examine the variables of general health on the SF36 and gender. In this analysis we had only two factors (F1 = 76.4%, F2 = 23.6%). Figure 5 shows the correlations between the variables gender and general health on the SF-36, and the co-occurrences of keywords. In the positive polarity of the X-axis we found the variables male (*V*-Test = 10.9511), lower scores on the SF-36 (*V*-Test = 10.9511), and lemma referring to the impact of medical therapies (Body, Cancer Treatment, Medical Operation, and Too Much). For the negative polarity of the ordinate we found the most significant categories to be female (*V*-Test = −10.9511), better scores on the SF-36 (*V*-Test = −10.9511), and keywords referring to people providing support in facing negative emotions during hospitalization (Mother, Sibling, Father Person, Donor, Anxiety, Attrition, Death). Regarding the negative polarity of the Y-axis, the results show that the categories male (*V*-Test = −6.0888) and better scores on the SF-36 (*V*-Test = −6.0888) were separated by the lemma To Transfer, Recovery, and Stress. Similarly, for the positive polarity the categories female (*V*-Test = 6.0888) and lower scores on the SF-36 (*V*-Test = 6.0888) were separated by the following lemma: Toy, Other Country, and Peers. The complete results are shown in Table 9.

**Table 9:** Lemma frequently used for Multiple Correspondence Analysis between General Health SF36 and Gender.

**F1 Test value**				**F2 Test value**			
**Pole (−)**	**Test-V**	**Pole (+)**	**Test-V**	**Pole (−)**	**Test-V**	**Pole (+)**	**Test-V**
*Female*	−10.95	*Male*	10.95	*Male*	−6.09	*Female*	6.09
*Sf36health_0*	−10.95	*Sf36health_1*	10.95	*Sf36health_0*	−6.09	*Sf36health_1*	6.09
To_Say	−3.33	Body	3.89	To_Transfer	−4.01	Toy	5.85
Person	−3.54	Cancer_Treatment	3.76	Recovery	−3.08	Other_Country	3.86
Donor	−3.54	Medical_Operation	3.01	Stress	−3.22	Peers	3.32
Mother	−3.87	Especially	2.87	Recover	−3.56	School	2.73
Anxiety	−2.06	Food	2.85	Until	−2.01	Job	2.70
Attrition	−2.06	Too_Much	2.70	Physical	−2.18	Many	2.62
Blood	−2.06	Due_To	2.68	Ward	−2.26	Because	2.61
Impossible	−2.06	Any	2.55	Period	−2.40	Volunteers	2.52
Sterilized	−2.09	Without	2.29	At_Last	−2.64	To_Play	2.46
Life	−2.18	Normal	2.07	To_Endure	−2.97	After	2.33
Room	−2.20	To_Begin	2.07			Experience	2.22
Sibling	−2.23					Deviant	2.21
Anyway	−2.28					To_Tell	2.21
Was_Were	−2.46					Don't_Understand	2.20
Death	−10.95					Solution	2.20
To_Arrive	−10.95					Yes	2.20
Psychologist	−3.33					Adjectives_Md_N^a^	2.20
Sick	−3.54					Age	2.18
Father	−3.54					To_Spend	2.18
To_Fight	−3.87					Too_Much	2.17
Md^b^	−2.06					All	2.02
Thanks	−2.06					Don't_Feel	2.01
To_Go	−2.06						

a *Positive adjectives to describe doctors and nurses*.

b *Medical Doctor*.

## Discussion

Hematopoietic Stem Cell Transplantation is a unique lifesaving therapy for some severe malignant and non-malignant diseases (Gratwohl et al., 2006; Ljungman et al., 2006). Although this therapy increases the possibility of survival and recovery from the primary disease, it also represents a stressor that in both the short and long term could affect all aspects of a patient's life, including social, cognitive, emotional, and behavioral functioning.

This exploratory study aimed to simultaneously investigate QoL and the psychological wellbeing of patients who underwent HSCT at pediatric age, in an approach combining both quantitative and qualitative methods taking into account the whole subjective experience.

The focus of the research was to reach a better, though not exhaustive, understanding of the possible conditions that survivors exposed to HSCT at pediatric age experience in adulthood with respect to QoL and psychological distress, and to investigate whether these domains present associations with specific medical and socio-demographic factors. The approach used provided not only a quantitative dimension to the investigated constructs but also the opportunity for the researchers to directly hear the subjective voice of those who have lived through the illness and the transplant experience, and thus to explore the process of elaborating on the trauma experienced at that time.

Given the pilot nature of the study and the small number of participants, it is possible that these results are not representative of the general population and should therefore be considered a descriptive and preliminary starting point from which to direct future research and stimulate clinical reflections. Moreover, only a small number of the subjects contacted agreed to participate in the study, thus further limiting the generalizability of the results.

Regarding the study participants, the sample appears well distributed with respect to gender, with the majority having reached a middle level of education. It may be worth noting that at the time of the study the average age of participants was 26 years, and that the majority were single and had an occupation. None of the subjects spontaneously requested psychological help. The majority of them reported that their memory of the information they had received about their medical and personal situation at the time was that it had been inadequate. It may be that this fact is somehow linked to our observation that the only difference between participants and non-participants was age at the moment of HSCT, with participants being younger. Indeed some authors have suggested that, compared with younger children, older children are more able to recall stressful procedures to mind and, as a consequence, are more likely to experience higher procedural anxiety connected with the event (Nuss and Wilson, 2007).

With regard to QoL, as hypothesized, participants reported greater difficulties concerning bodily pain, general health perception, and physical and social functioning, while experiencing better functioning in the other domains. This result is in line with the findings of other studies suggesting that although patients may report a good QoL, it is impaired in comparison with that of the general population (Hjermstad et al., 2004). HSCT recipients face numerous physical challenges before, during, and after the medical course, and various studies have pointed to the strong associations that exist between transplant-related distress and QoL (Hjermstad et al., 2004; Andrykowski et al., 2005; Lee et al., 2005). In line with this, we found that the aspects that were more compromised were those in which the body is involved. Moreover, these results support the hypothesis that physical limitations on activities as a result of medical procedures can significantly affect patients' social relationships and social functioning (Vannatta et al., 1998). Given that the majority of participants declared themselves single, it is reasonable to speculate on whether there are some associations between these impairments in social functioning and the absence of a partner. Furthermore, some authors have suggested that HSCT survivors are likely to experience a decline in intimacy due to alterations in their sexual functioning and satisfaction, which can detract from their ability to build a solid and stable relationship (Tierney, 2004).

As for the SCL-90-R scores, survivors did not report clinically significant distress affecting their global psychological status, as evidenced by the small number of subjects above the GSI cut-off. HSCT survivors more frequently experienced difficulties in specific domains, such as sleep, interpersonal sensitivity, and obsessive-compulsive symptoms. Difficulties relating to interpersonal sensitivity and obsessive-compulsive symptoms have been reported in the literature (Wiener et al., 2006; Çuhadar et al., 2016), although not that often. Both of these symptom categories deal with the anxiety-depression area, as well as sleep disorders. One possible hypothesis for our findings could be that the experience of HSCT has been so emotionally intense that patients still find it difficult to get over it. As a consequence, the high arousal associated with this memory may lead to the persistence of post-traumatic stress-like symptoms, which are well documented in several studies, long after the HSCT event (Jacobsen et al., 2002; DuHamel et al., 2004; Lee et al., 2005).

As far as the effects of medical and socio-demographic factors are concerned, we found that specific variables were shown to be associated with QoL and psychopathology.

As for demographic variables, male subjects reported a higher degree of symptoms on the SCL-90-R scales and lower scores on the SF-36, suggesting that female survivors are more likely to experience better QoL and less psychological distress in contrast to what was expected. Similar results have been reported in other studies, suggesting that better perception of QoL might be associated with positive social support and positive relationships with others (Andrykowski et al., 2005; Corey et al., 2008; Sundberg et al., 2009; Enskär and Berterö, 2010); it was suggested that women might experience higher QoL and lower psychopathological symptoms due to their ability to build stronger bonds with family and friends (Enskär and Berterö, 2010). Females are usually more prone both to share emotions and to express them, being more capable than males of providing and receiving emotional support (Turner, 1994). This also seems to be confirmed by the T-lab analysis. In their reports, females talked of other human subjects sharing their experience, such as family members and medical staff. Females also reported more positive interaction with others, as confirmed by Enskär and Berterö (2010) and Sundberg et al. (2009); this could constitute a protective factor such that the perceived support of friends, family, and healthcare providers is associated with less depression and anxiety (Corey et al., 2008). On the other hand, in the texts of males, recurring lemma showed no human references but focused on medical aspects of therapy, things they were obliged to do, and the feeling of being without something they would like. It may be that the presence of relational support provided by different people, such as volunteers, could represent a protective factor, as opposed to the impossibility to understand and to feel, as reported by the males.

Turning to the medical variables, the type of pathology, the type of HSCT, the time elapsed between communication of the need to transplant and effective transplantation, and the days of hospitalization in HSCT Unit and after the procedure, were shown to be associated with psychopathology and QoL. More specifically, compared with patients affected by leukemias or other diseases, survivors of solid tumors reported higher scores on the SCL-90-R.

Although we hypothesized more difficulties in patients who had undergone allogeneic HSCT, our results actually revealed that survivors who had undergone autologous transplantation experienced higher psychological symptoms and lower QoL. This was due to role limitations resulting from emotional problems and difficulties in social functioning. Other authors have previously shown correlations between autologous transplantation and the perception of lower physical functioning (Sanders et al., 2010). One possible explanation for this finding is the possible persistence of risk in these patients, having received their own cured stem cells rather than originally healthy cells.

Psychopathology was also shown to be associated with the time elapsed between communication of the need to transplant and effective transplantation, with patients who had waited between 6 and 12 months reporting higher scores on interpersonal sensitivity, depression, psychoticism, paranoid ideation, phobic anxiety, and sleep problems. Patients who had waited less than 6 months did not present such problems, nor did patients who had waited more than 1 year. The period between 6 and 12 months appears to be a more delicate time: patients probably perceived the increasing waiting time as a risk, being insufficiently stable to be out of danger.

Length of hospitalization both in the HSCT Unit and after transplantation was shown to be associated with phobic anxiety and depression. It is possible that the increase in time passed in hospital, associated with the sequence of medical practices, enhances an individual's fears for his/her physical wellbeing, engendering feelings of anxiety and distressing thoughts about contamination, disease relapse, and the need for control. Moreover, it has to be wondered whether the strong limitations on activities and social relationships following prolonged hospitalization could be associated with a decline in mood.

Finally, males who underwent an autologous transplant after waiting 6–12 months from HSCT communication to the procedure itself appear to be the most fragile group of patients. Therefore, more attention should be paid to the arrangement of adequate time and settings when providing support, so that children can be listened to and helped, even in such a difficult place as an oncological unit.

Qualitative analysis using T-Lab permitted us to detect the main themes linked to transplant experience reported by a subgroup of the sample. As shown in Figure 1, the distribution of lemma and their co-occurrences in the Cartesian plane seem to show spatially defined thematic areas.

The first area considered (+X, +Y) groups memories and forgetfulness of hospital life, medical therapies, recoveries, acute complications of transplant, and disease in general. The only reference to people is to other sick children. The second area (+X, −Y) seems to be associated with precise moments and memories lived in the isolation of the hospital ward; it concerns elapsed time between medical operations, and examinations or cancer treatments such as chemotherapy or radiotherapy, the impact of which have left traces in terms of bodily changes.

Both areas contain themes about the physical aspects of therapy and of the body, suggesting that these aspects can affect memories even at a distance of time. The dimension of the body seems to recur in every tool used in the entire study, pointing to the importance of dedicating enough space to body issues during clinical practice and strengthening environmental support in terms of spaces dedicated to play and family.

In the negative areas of the X-axis, affections and relational aspects connected to people were the main ones to emerge. In the quadrant (−X, +Y) there are contents dealing with a sort of remote memory, dominated by the fact that today it is possible to remember difficulties as well as the good fortune to be alive; this includes remembering hospital staff, and ascribing positive adjectives to doctors and nurses. The last quadrant (-X, -Y) focuses on family members, and on the fatigue and suffering that all of them had to face.

The concept of luck seems to be retrospectively linked to the fact of surviving and to familial support. The suffering remembered seems to be mainly emotional-psychological: medical care seems to provide a response to the disease, so the painful memory maintained is mainly psychological.

The T-Lab analysis appears to suggest a separation between two main aspects of the disease and the care: one more concrete and one more relational concerning inherent feelings. Examining the qualitative data, there seems to be a split between body and mind that persists in memories even at a distance: there are some memories about medical body procedures and others linked to feelings and interpersonal relationships. The only feeling that emerges in the positive area (concrete aspects) is the fear (at the boundaries of the areas), strongly linked to disease; other feelings are linked to relationship (negative areas of the axis). It could be that this kind of split is in some way functional, to provide protection from the trauma, as supported by the normal values on the mental health scale of the SF-36.

Traumatic aspects seem to be linked to concrete and physical issues and the whole experience could be tolerated when both positive and negative feelings are expressed in a “relational narration.” This seems to be in accordance with Lehmann et al. (2014), who highlighted how bodily concerns and the difficulties experienced when interacting with others constitute two of the negative consequences still reported by survivors even 10 years after cancer diagnosis.

Regarding perception of the disease and therapy, it is interesting to consider the differences between the genders: for example, females refer more frequently to human subjects, family members or others, while males strongly focus on the entire medical therapy, such as cancer treatments, medical operations, medical exams, and drugs, and also on negative aspects such as stress and fears. According to (Enskär and Berterö, 2010) and Sundberg et al. (2009) and in accordance with the results of the other tools described above, relationships for female subjects could represent a protective factor for better QoL, reduced psychopathology, and ultimately resilience.

As for the disease treated and the kind of transplantation, leukemias were linked to allogeneic HSCT from an unrelated donor while the most recurrent lemma were ones concerning subjects in an impersonal way or clearly identified as parents. These words correlated with aspects of the care, the disease, the donor, the salvation, toys, and the act of playing. References to relationship were clearly linked to the fact that healing was achieved through another subject (the donor). Moreover, patients treated by allogeneic transplant from an unrelated donor presented a better perception of general health on the SF-36, in line with the prognosis generally associated with this type of HSCT. At the same time, other semantic areas that directly concerned the disease, such as death, anxiety, recoveries, and the ward, also found a space in the compositions.

The second category of underlying diseases (non-Hodgkin's lymphoma, neuroblastic tumor, Ewing's sarcoma, Hodgkin's Disease), more closely linked to autologous transplantation, was mostly connected to negative and constrictive aspects, stress, recoveries, drugs, and medical therapies. According to these results, these patients seem more likely to concentrate their memories on everyday ward life and on themselves.

Adults who had received an autologous transplant also showed worse scores on the SF-36 subscale which, according to Sanders et al. (2010), indicates how this kind of transplant is a risk factor for worse physical functioning. In this group, the main lemma focused on cancer treatments, radiotherapy, and medical operations, all of which refer to medical aspects involving the body that could be perceived as “too much.” This is in contrast with the other group, which refers to the body in terms of aspects associated with physical wellbeing. The only human figures to emerge here were friends, in terms of differentiating these children's lives from those of their peers.

Finally, the last group of diseases (Cooley's anemia, Fanconi anemia, and Mucopolysaccharidosis), linked to allogeneic HSCT from a related donor, produced references to the travel to Padua from other countries. There were also references to temporal distance, indicating that today it is possible to consider the pain, faith, and possibility of success. Moreover, patients belonging to this category mostly referred to hair loss and in general felt more different compared with peers, probably due to the chronic nature of their underlying disease. Spatial and temporal dimensions were present here, probably because Padua represents a reference center for this rare group of diseases.

As mentioned previously, the present study has various limitations that prevent the generalization of its results but which offer useful suggestions for future research. One of its main limitations is the small number of participants. The fact that only 13% of initially eligible survivors agreed to participate is particularly surprising. The reasons for this extremely low rate varied, ranging from the difficulty of contacting subjects because they had relocated or changed phone number, to their overt wish to forget the period of their HSCT, suggesting perhaps, that even when far removed from the intervention itself the experience of the transplantation elicits intense emotions that may impede recall of the event. Further research is needed to better clarify this aspect and to investigate whether there are specific factors that might be associated with this difficulty.

Moreover, some of the survivors did not have fixed check-ups during the research period, or had no more follow-ups at the clinic. This finding raises questions about the need to find the best possible strategies for maintaining contact with survivors exposed to HSCT and carry out preventive long-term psychological screening. Different authors have pointed out that although clinical research plays an important role in enhancing supportive care and understanding of disease risks (Ferrara et al., 2007; Gooley et al., 2010), one of the biggest challenges is that only a few patients ever enter into research protocols (Umutyan et al., 2008; Keusch et al., 2014). Furthermore, only a few studies have explored barriers to participation specific to HSCT research, suggesting the need to help participants to fully comprehend the implications of taking part in these studies (Denzen et al., 2012; Raich et al., 2012; Keusch et al., 2014). At the same time, the importance of helping patients to distinguish between research and routine care activity so that they do not risk feeling confused or overwhelmed by the request to provide the same information on multiple forms, has been pointed out (Keusch et al., 2014). Some authors have suggested that a useful way to meet this objective might be to extend discussions between clinicians, researchers, and potential research participants (Aaronson et al., 1996).

Despite the small number of participants, one of the main strengths of the present study is the use of a combined quantitative and qualitative approach. Only a few studies have investigated the long-term consequences of HCST in a qualitative way and with specific reference to subjective experience (Beeken et al., 2011; Lahaye et al., 2017). Integration between the values obtained using a standardized tool and a specific focus on narrations can allow one to reach a complete understanding of the different dimensions of the experience. More specifically, the adoption of such an approach allowed us to reveal the subjective experience behind the standard categories offered by tests.

This combined information can be translated into clinical practice: on the one hand, to improve the clinician's relationship with the patient and his/her compliance with medical treatment, in line with more recent approaches in narrative ethics that underline how narrations can help to improve the process of care (Frank, 2016); and on the other, to orientate intervention during all the phases that precede, accompany, and follow HSCT.

In conclusion, the results of the study appear to be in line with previous empirical research in showing that survivors who undergoHSCT during childhood are at higher risk of psychological distress and poor QoL in the long term. Moreover, these difficulties appear to be exacerbated by the presence of specific socio-demographic and medical factors that should, therefore, be considered when planning patient-tailored treatment. The early detection of such aspects could consequently help to identify at an early stage those subjects to whom clinicians should pay more attention when providing services and support, both during HSCT and once the process of reintegration to normal routines following transplantation has begun.

## Author contributions

FS: Wrote the manuscript, data analysis, data interpretation. AT: co-wrote the manuscript, data interpretation. SZ: Data acquisition; Co-wrote the manuscript, data interpretation. ND: Co-wrote the manuscript, data management. AP: co-wrote the manuscript, data interpretation. MT: Data analysis. VB: Data acquisition. AM: Medical data collection. CMa: Helped in medical data collection, in reviewing the literature. EC: Medical data collection. MP: Medical data collection. CC: Designed the work, supervision of data collection, critical revision of the work. GB: Revising work, coordinator of the intervention, helped in designing research project CMe: Designed the work, coordinator of the intervention.

### Conflict of interest statement

The authors declare that the research was conducted in the absence of any commercial or financial relationships that could be construed as a potential conflict of interest.

## Footnotes

^1^IBM SPSS statistics version 23. Armonk, NY: IBM Corporation.
